# Regulation of TrkB cell surface expression—a mechanism for modulation of neuronal responsiveness to brain-derived neurotrophic factor

**DOI:** 10.1007/s00441-020-03224-7

**Published:** 2020-06-15

**Authors:** Thomas Andreska, Patrick Lüningschrör, Michael Sendtner

**Affiliations:** Institute of Clinical Neurobiology, University Hospital Wuerzburg, 97080 Wuerzburg, Germany

**Keywords:** BDNF, TrkB, Subcellular trafficking, Transactivation, Synaptic plasticity

## Abstract

Neurotrophin signaling via receptor tyrosine kinases is essential for the development and function of the nervous system in vertebrates. TrkB activation and signaling show substantial differences to other receptor tyrosine kinases of the Trk family that mediate the responses to nerve growth factor and neurotrophin-3. Growing evidence suggests that TrkB cell surface expression is highly regulated and determines the sensitivity of neurons to brain-derived neurotrophic factor (BDNF). This translocation of TrkB depends on co-factors and modulators of cAMP levels, N-glycosylation, and receptor transactivation. This process can occur in very short time periods and the resulting rapid modulation of target cell sensitivity to BDNF could represent a mechanism for fine-tuning of synaptic plasticity and communication in complex neuronal networks. This review focuses on those modulatory mechanisms in neurons that regulate responsiveness to BDNF via control of TrkB surface expression.

## Introduction

Nerve growth factor (NGF) was the first member of the neurotrophin family that was discovered as a survival factor for distinct populations of neurons during a critical period of development. When brain-derived neurotrophic factor (BDNF) and the other members of the neurotrophin family were identified, it became apparent that the responsiveness of BDNF-sensitive neurons was also qualitatively different from NGF-dependent neurons. These differences can be explained by differential target cell responsiveness to BDNF rather than differential expression of the corresponding TrkB receptor. In the present article, we aim to provide an overview about some historic aspects that first pointed to differences between NGF and BDNF, and recent findings on molecular mechanisms how target cell sensitivity to BDNF signaling is regulated by TrkB cell surface expression. These mechanisms could explain differential responsiveness of developing neurons to NGF and BDNF.

Work by Viktor Hamburger and others has shown that many types of neurons are generated in excess in the embryonic nervous system of vertebrates and that only subpopulations that are supported by neurotrophic factors are maintained (reviewed by Purves and Lichtman 1985a). In the peripheral nervous system, these neurotrophic factors have been proposed to be derived from innervated target areas (reviewed by Purves and Lichtman 1985a), based on observations such that limb bud elimination during this critical period enhances cell death (Hamburger 1934). At the same time, augmentation of target tissue by transplantation of a supernumerary limb reduces neuronal loss. This was the basis for the discovery of nerve growth factor (NGF), the first prototypical neurotrophic factor (reviewed by Lewin and Barde 1996, Purves and Lichtman 1985b). Neurotrophins mediate survival effects via specific transmembrane tyrosine kinase receptors of the Trk family, TrkA, TrkB, and TrkC (reviewed by Chao 2003; Huang and Reichardt 2003; Kaplan and Miller 2000). NGF is required for the survival of developing sensory and sympathetic neurons (Thoenen et al. 1981) but not spinal motoneurons.

Whereas the physiological actions of NGF on developing sympathetic and sensory neurons were confirmed by analyses of in vitro models and knockout mice (Crowley et al. 1994; Nikoletopoulou et al. 2010; Ruberti et al. 2000), the physiological action of BDNF on developing spinal motoneurons remained obscure and partially also controversial. While isolated early sensory ganglionic neurons from E8 chick embryos survive in the presence of NGF (Davies and Lindsay 1985; Edgar and Thoenen 1982; Thoenen et al. 1981), E6 chick spinal motoneurons can be maintained in cell culture only at low efficacy with BDNF (Arakawa et al. 1990). As a positive control, other neurotrophic factors such as CNTF appeared much more potent (Arakawa et al. 1990; Berkemeier et al. 1991). However, responsiveness to BDNF developed in cultures of embryonic chick motoneurons when these cells were primed with muscle extracts (Becker et al. 1998). Subsequent studies showed that it was not the absence of TrkB that was responsible for the low survival response of early embryonic chick motoneurons to BDNF. TrkB was abundantly expressed in early chick motoneurons and it appeared activated even in the absence of BDNF when these cells were treated with glial-derived neurotrophic factor (GDNF), ciliary neurotrophic factor (CNTF), or fibroblast growth factor (FGF) (Becker et al. 1998). This indicates that muscle extracts contain factors similar to CNTF or FGF that potentiate the response of developing motoneurons to BDNF. This could be interpreted as a differentiation effect that activates responsiveness to BDNF. Alternatively, and not exclusively, other growth factors also could transactivate TrkB (Castellino and Chao 1996; Lee and Chao 2001), and the survival-promoting effects of activated G protein–coupled receptors such as adenosine type 2A receptors (A_2_A-R) depend on the presence of TrkB (Wiese et al. 2007). On the other hand, isolated rat or mouse motoneurons from E12 to E15 embryos survive with high efficacy in the presence of BDNF when cultured with enriched cell culture media (Hughes et al. 1993). Moreover, postnatal motoneurons can be protected from axotomy-induced cell death by application of BDNF (Sendtner et al. 1992; Yan et al. 1992), and this effect was also observed in embryonic chick after limb bud removal (Oppenheim et al. 1992). These findings suggested that the TrkB-mediated response of developing motoneurons to BDNF undergoes alterations during development and might differ from the prototypic response of embryonic sympathetic or sensory neurons to NGF. This hypothesis is also supported by findings from NGF (Crowley et al. 1994; Ruberti et al. 2000) and BDNF (Liu et al. 1995) gene knockout mice. Whereas NGF knockout mice show massive developmental cell death of sympathetic or sensory neurons (Crowley et al. 1994; Ruberti et al. 2000), no enhanced loss of motoneurons could be observed in BDNF and NT-4 knockout mice (Liu et al. 1995), indicating that the dependency and response of developing sensory and motoneurons to neurotrophins differ. A difference in response of TrkA- and TrkB-expressing neurons became also apparent from experiments with stem cell–derived neurons showing that TrkB, unlike TrkA and TrkC, does not induce cell death in absence of its ligand (Nikoletopoulou et al. 2010). This implies that BDNF responsiveness, in contrast to the response to NGF or NT-3, is a highly regulated process that depends on co-factors and modulators. This review focuses on those modulatory mechanisms in neurons that regulate responsiveness to BDNF.

## Regulation of TrkB cell surface expression

First insights into the mechanisms that modulate responsiveness to BDNF in developing neurons came from analyses with cultured rat retinal ganglion cells (RGCs) and spinal motoneurons (Meyer-Franke et al. 1995; Meyer-Franke et al. 1998). These cultured neurons show only low responsiveness to BDNF despite robust TrkB expression levels. This low responsiveness was found to be due to low levels of TrkB at the cell surface when these neurons were cultured under serum-free conditions (Meyer-Franke et al. 1998). cAMP was identified in these studies as a key modulator of TrkB cell surface expression. This second messenger strongly induces responsiveness to BDNF in these neurons (Meyer-Franke et al. 1998). Previous studies had shown that cAMP also increases the responsiveness to other neurotrophins such as NGF in adrenal medullary derived neuronal cell lines (Birren et al. 1992). However, the effect of cAMP in these sympathoadrenal precursor cells was due to a transcriptional upregulation of TrkA expression and thus differed from the upregulation of cell surface translocation that had been observed for other transmembrane proteins such as ion pumps or transporters in non-neuronal cells (Barres et al. 1989; Lewis and de Moura 1984; Li et al. 1982; Murer and Biber 1996; Schwartz and Al-Awqati 1986; Wade 1986; Yao et al. 1996). In contrast, NT-3 mediated survival and TrkC activation in hippocampal neurons appeared independent of cAMP treatment (Ji et al. 2005). Experiments using *Xenopus* nerve-muscle co-cultures and rat hippocampal slice cultures showed that BDNF responsiveness is potentiated by cAMP analogs, while inhibition of cAMP signaling reduces TrkB phosphorylation, and cAMP alone cannot mimic the neurotrophin effects on synaptic potentiation (Boulanger and Poo 1999; Tartaglia et al. 2001). Depolarization by high K^+^ levels or electrical stimulation has a similar effect as cAMP treatment. It activates Ca^2+^-dependent adenylyl cyclases via Ca^2+^ influx through ion channels and NMDA receptors (Du et al. 2000; Xia et al. 1991). This also results in elevated cAMP levels and increased BDNF responsiveness (Meyer-Franke et al. 1995; Meyer-Franke et al. 1998). Two characteristic parameters indicate that this cAMP-induced BDNF responsiveness is due to a rapid TrkB surface transport from pre-existing intracellular pools rather than de novo transcription or translation of the mRNA for this receptor. First, BDNF responsiveness occurs in a relatively short time course of less than 5 min (Hanson Jr. et al. 1998). Second, inhibition of translation with cycloheximide is unable to block cAMP-mediated BDNF responsiveness (Cheng et al. 2011; Du et al. 2000; Meyer-Franke et al. 1998; Zhao et al. 2009). Furthermore, pretreatment with nocodazole or cytochalasin D prevents TrkB translocation to the cell surface, thus providing proof that cAMP-induced TrkB translocation depends on intact microtubule and actin microfilament architecture (Zhao et al. 2009).

The cAMP-mediated induction of BDNF responsiveness was observed also in other cell types such as cortical neurons (McAllister et al. 1996). cAMP itself acts in a pathway with protein kinase A (PKA) and phosphoinositide 3-kinase (PI3K), and inhibition of either kinase drastically reduces the viability of cultured retinal ganglion cells and spinal motoneurons (Hanson Jr. et al. 1998; Meyer-Franke et al. 1995; Meyer-Franke et al. 1998). This treatment also reduces TrkB surface expression in hippocampal neurons (Cheng et al. 2011). Similarly, PI3K activity has been shown before to mediate the effects of insulin on cell surface expression of transient receptor potential cation channel (TRPV2) in Min6, CHO, and dispersed pancreatic beta-cells (Hisanaga et al. 2009). This observation revealed parallels to cAMP-induced TrkB surface translocation since it requires depolarization via Ca^2+^ entrance and an intact cytoskeleton, especially intact actin filaments (Hisanaga et al. 2009). In conclusion, cAMP was proposed to have a “gating” function that elevates translocation of TrkB via PKA and PI3K activity from intracellular stores to the cell membrane (Cheng et al. 2011; Du et al. 2000; Ji et al. 2005; Meyer-Franke et al. 1998; Zhang et al. 2000; Zhao et al. 2009).

## Dynamics of TrkB cell surface translocation

TrkB cell surface translocation is accompanied by a significant enrichment of TrkB in postsynaptic spines, thus leading to a rapid enhancement in sensitivity for incoming BDNF signaling (Ji et al. 2005; Sui et al. 2015; Zhao et al. 2009). This allows BDNF-mediated short-term changes in synaptic transmission which then are further strengthened by dendritic growth and structural refinement of synapses in an activity-dependent manner (Lohof et al. 1993; Lu 2004; McAllister et al. 1999; Wang et al. 1995). In fact, BDNF stimulation increases synapse numbers in hippocampal and cerebellar neurons (Shimada et al. 1998; Tyler and Pozzo-Miller 2001) and also modulates dendritic complexity especially in the striatum (Rauskolb et al. 2010) and hippocampus (Tyler and Pozzo-Miller 2001). Association of TrkB with postsynaptic spines was further confirmed by co-localization and co-immunoprecipitation with PSD-95 which modulates guidance and anchoring of transmembrane receptors and ion channels into dendritic spines (Ji et al. 2005; Scannevin and Huganir 2000; Sheng 2001; Zhao et al. 2009). Interestingly, the dynamics of cAMP-mediated TrkB translocation and localization in dendritic spines after chemically induced LTP (cLTP) (Zhao et al. 2009) was described to parallel AMPA receptor surface translocation under similar conditions (Yudowski et al. 2007).

BDNF secretion and local recruitment of TrkB are also crucial elements for axon differentiation (Cabelli et al. 1995). BDNF itself acts in a self-amplifying manner by promoting TrkB surface expression through elevation of cAMP and thereby drives differentiation of neurites towards axonal fate (Cheng et al. 2011; Shelly et al. 2007; Shelly et al. 2011). Thus, the differentiation of axons and dendrites seems to be controlled by redistribution of cAMP-/PKA-sensitive autocrine BDNF secretion and TrkB surface expression towards the axon (Cheng et al. 2011). Local changes in cAMP levels and PKA activity can induce axon formation in rat hippocampal neurons by modulating LKB1 and SAD kinases (Barnes et al. 2007; Kishi et al. 2005; Shelly et al. 2007). Such local cAMP changes could also fine-tune individual axonal terminals, as shown in *Drosophila* motoneurons (Maiellaro et al. 2016). This could be important for neurons with high numbers of axonal terminals, such as spinal motoneurons because it allows autonomic regulation of BDNF responsiveness at each individual axon terminal within the same neuron. Similarly, BDNF responsiveness could be regulated by TrkB surface translocation into individual dendritic spines, a process that was observed in cultured hippocampal neurons (Ji et al. 2005).

**Fig. 1:**
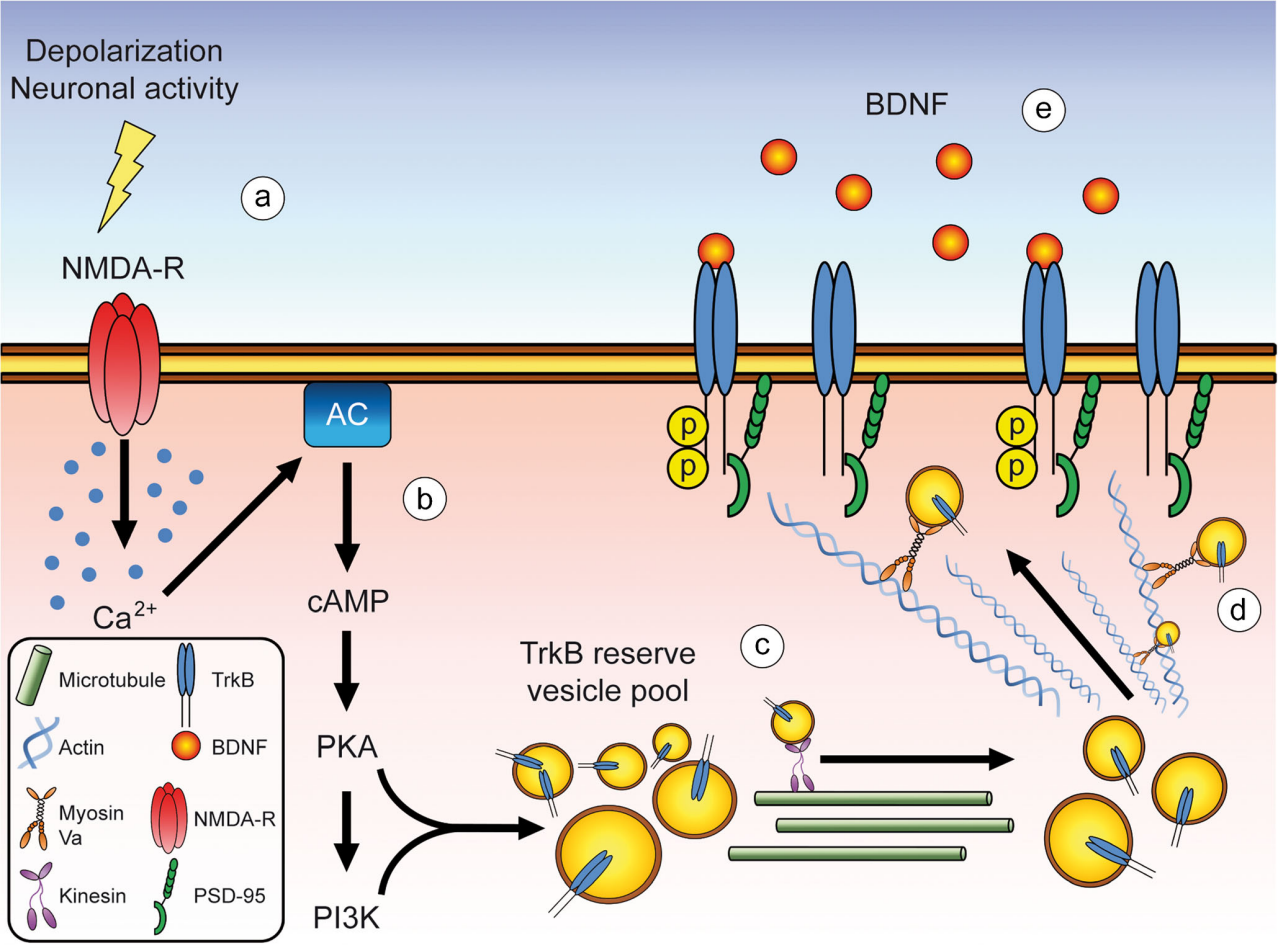
Schematic representation of cAMP-mediated TrkB surface translocation via PKA/PI3K. Neuronal activity triggers Ca^2+^ entry via AMPA and NMDA receptors (a). Ca^2+^ stimulates adenylyl cyclase activity followed by elevation of cAMP levels which trigger PKA and PI3K activity (b). Rapidly available intracellular reserve pools of TrkB are mobilized in a microtubule-dependent manner (c). TrkB containing vesicles are transported to distinct target sites at the cell surface, like dendritic spines where they integrate. In the dendritic target area, TrkB is transported via Myosin Va in an actin-dependent manner and associates with PSD-95 which promotes guided transport and integration into the PSD (d). At the cell surface, TrkB is sensitive for BDNF binding which activates TrkB via phosphorylation at C-terminal tyrosine residues (e)

In summary, these studies provide evidence that cAMP-mediated TrkB surface translocation in dendritic spines but also axon terminals involves several critical steps **(**Fig. 1**)**. First, neuronal activity triggers Ca^2+^ entry via AMPA and NMDA receptors (a). Ca^2+^ stimulates adenylyl cyclase activity, thus causing an elevation of cAMP levels which trigger PKA and PI3K activity (b). This leads to the mobilization of a rapidly available intracellular reserve pool of TrkB (c). TrkB containing vesicles are transported in a microtubule-dependent manner to distinct sites at the cell surface where they integrate, such as dendritic spines. In the dendritic target area, TrkB is transported in an actin-dependent manner via Myosin Va and associates with PSD-95 for guided integration into the PSD (d). TrkB is then able to bind BDNF and gets activated via autophosphorylation at C-terminal tyrosine residues (e).

## The role of G protein–coupled receptors in TrkB signaling

Elevated levels of cAMP play a central role in the translocation of TrkB to distinct cell surface regions within neurons. Thus, it is not surprising that G protein–coupled receptors (GPCRs) were proposed as key activators and potential central physiological regulators for the effects observed in cultured neurons after cAMP treatment (Ji et al. 2005; Luttrell 2008).

G protein–coupled receptors (GPCRs) constitute the largest and most diverse family of transmembrane receptors (for review see (Huang and Thathiah 2015; Luttrell 2008)). Intracellular signal transduction mediates cell proliferation, growth, differentiation, and neurotransmission including synaptic plasticity through modulation of presynaptic and postsynaptic architecture and transmitter release (Gainetdinov et al. 2004; Huang and Thathiah 2015; Luttrell 2008). Ligand binding to GPCRs causes dissociation of G_α_ subfamily members of G proteins which modulate the activity of effector enzymes like adenylyl cyclases (AC), phospholipase C (PLC), or L- and N-type Ca^2+^ channels (Luttrell 2008). GPCRs can change the concentration of second messenger molecules like cAMP via this G_α_ signaling pathway and thus modulate PKA activity (Luttrell 2008). In parallel, released G_βγ_ subunits modulate PKC activity and Ca^2+^ release from the endoplasmatic reticulum (ER) via stimulation of phospholipase C-beta (PLC-β) activity (Clapham and Neer 1993; Morris and Scarlata 1997; Yan et al. 1999). Due to the ability to change cAMP and Ca^2+^ levels, GPCRs potentially modulate TrkB surface translocation. Early evidence supporting this hypothesis has been provided by studies using cultured striatal medium spiny neurons (MSNs) (Du et al. 1995). This work showed that dopamine addition potentiated the effects of BDNF on the differentiation of tyrosine hydroxylase–positive striatal neurons. This finding was supported by subsequent studies both in vivo and in vitro (Li et al. 2012; Plotkin et al. 2014). Taken together, these studies suggested that the sensitivity of striatal neurons to BDNF depends on a “co-factor”. In particular, G_αs_–coupled GPCRs which elevate cAMP levels were found to promote corticostriatal LTP, a mechanism which most likely also involves BDNF/TrkB signaling (Plotkin et al. 2014; Shen et al. 2008, 2016; Zhai et al. 2019). Indeed, LTP of corticostriatal synapses was found to depend on coordinated activation of NMDARs, TrkB, and G_αs_–coupled GPCRs like DRD1 or adenosine type 2A receptors (A_2_A-R) (Plotkin et al. 2014; Shen et al. 2008, 2016; Zhai et al. 2019). However, the precise role of TrkB in this context remained undefined. Stimulation of G_αs_–coupled DRD1 can elevate TrkB surface expression in mixed cultures of striatal neurons (Iwakura et al. 2008) after periods of several hours. However, it remained unclear whether the increased cell surface expression was due to enhanced expression on the transcriptional and/or translational level, or enhanced cell surface translocation from pre-existing intracellular pools. It is tempting to speculate that the effects of D1 receptors on striatal LTP (Plotkin et al. 2014; Shen et al. 2008, 2016; Zhai et al. 2019) and the effects of D2 receptor stimulation on striatal LTD (Kreitzer and Malenka 2005; Shen et al. 2008, 2016; Zhai et al. 2019) involve alterations in BDNF/TrkB signaling. However, the mechanisms for this effect are still unresolved. In particular, the question remains to be determined whether alterations in transcriptional or translational control or rapid effects on subcellular transport are responsible. The relatively long time course of several hours observed for altered TrkB surface expression after D1 stimulation in cultured striatal neurons (Iwakura et al. 2008) suggests that transcriptional and translational controls are involved. However, this stands in contrast to the rapid effects of enhanced BDNF responsiveness within minutes after treatment of hippocampal neurons with agonists for dopamine or adrenergic receptors (Ji et al. 2005). If the hypothesis that GPCR signaling acts as “co-factor” to promote BDNF sensitivity via TrkB surface translocation is correct, one would also expect that ablation of this “co-factor” has similar consequences as ablation of BDNF or TrkB. Dopamine depletion is known to cause massive spine loss on striatal neurons (Day et al. 2006; Deutch 2006; Deutch et al. 2007; Gerfen 2006; Ingham et al. 1989; Ingham et al. 1998; McNeill et al. 1988; Villalba et al. 2009; Villalba and Smith 2010; Zaja-Milatovic et al. 2005) and altered LTP/LTD (Plotkin et al. 2014; Shen et al. 2008, 2016; Zhai et al. 2018). Similarly, conditional postnatal depletion of BDNF (Li et al. 2012; Rauskolb et al. 2010) or TrkB (Baydyuk et al. 2011; Li et al. 2012) also causes decreased striatal volume, dendritic atrophy, and spine loss in striatal MSNs, while scavenging of striatal BDNF suppresses corticostriatal LTP (Jia et al. 2010).

## The role of N-glycosylation in cargo translocation from the ER

**Fig. 2:**
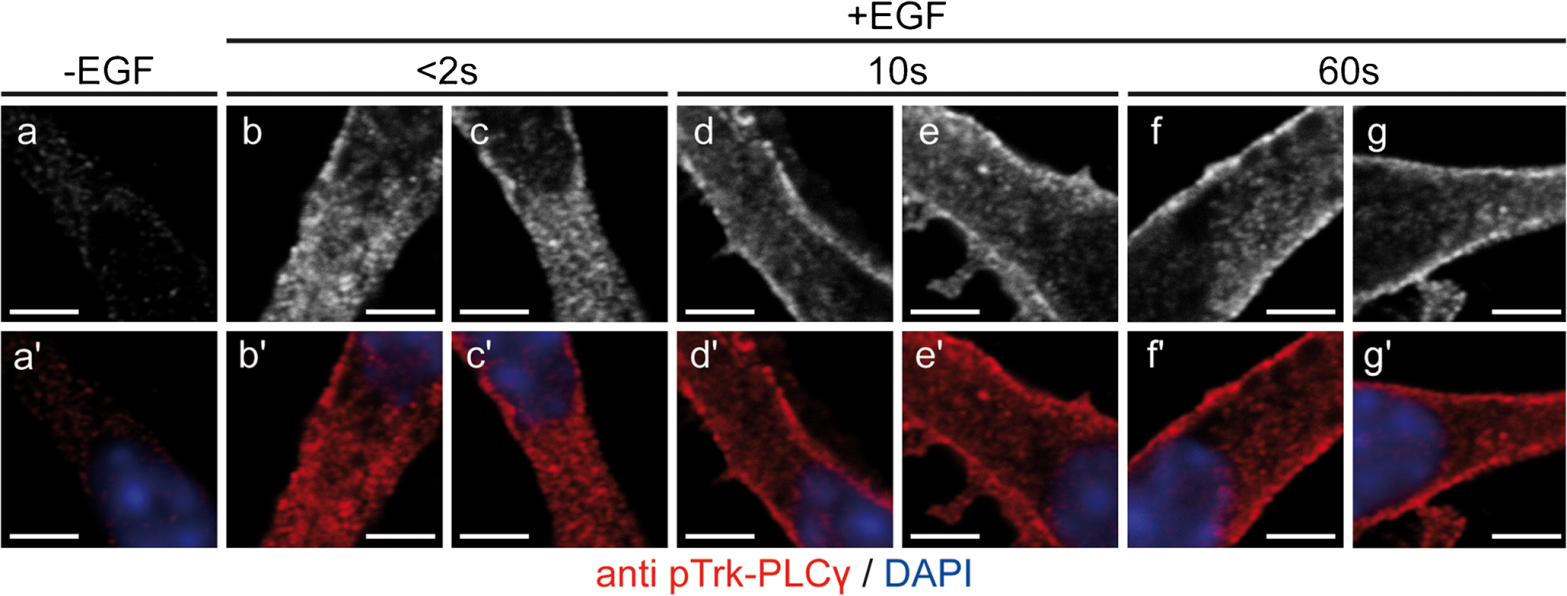
Rapid TrkB surface translocation upon EGF stimulation of cortical precursor cells. Time course of pTrk-PLCγ activation after EGF stimulation in cortical precursor cells (a–g: pTrk-PLCγ-IR; a′–g′: merged images). Immediately after the EGF pulse, after less than 2s (b, b′), the signal for pTrk-PLCγ appears first intracellularly and subsequently at the cell membrane after 10s (d/d–-g/g′). Bar: 3 μm. Figure reproduced with permission from Puehringer et al. Nature Neurosci. 2013

Apart from the ability to modulate TrkB surface translocation, GPCRs are also known to activate Trk receptors in the absence of neurotrophins, a process called transactivation (Iwakura et al. 2008; Lee and Chao 2001; Wiese et al. 2007). Similar transactivation effects were also observed in early cortical neurons when these cells were treated with epidermal growth factor (EGF) via activation of the EGF transmembrane tyrosine kinase receptor (EGFR) (Puehringer et al. 2013). TrkB plays a critical role for the migration of these early neurons (Bartkowska et al. 2007; Medina et al. 2004) at developmental stages when endogenous BDNF is still not expressed (Maisonpierre et al. 1990; Puehringer et al. 2013). This raises the question of how TrkB is activated in this developmental context. Phosphorylation of TrkB is not reduced in E12 brain of BDNF/NT-3 double knockout mice. Instead, a massive reduction of TrkB activation was observed in EGFR KO brain at this stage (Puehringer et al. 2013). In the same early cortical neurons, TrkB transactivation by EGFR signaling was also found to drive TrkB translocation from intracellular stores to the cell surface (Puehringer et al. 2013). This endogenous TrkB appeared to be recruited from the endoplasmatic reticulum to the cell surface within a few seconds (Fig. 2). Inhibition of N-glycosylation disturbed this process by abolishing retention of TrkB in the ER. This indicates that TrkB N-glycosylation plays a central role in the rapid recruitment and translocation of this receptor from the ER to the cell surface.

Glycan structures on newly synthesized glycoproteins are crucial for protein secretion. N-glycosylation is the most frequent form of posttranslational modification in the ER (Moremen et al. 2012). During this process, glycans are transferred from a lipid-linked oligosaccharide to the nascent polypeptide chain during translation (Moremen et al. 2012). After this initial transfer, trimming of two glucose residues and sequential addition of diverse monosaccharides like glucose (Glc), *N*-acetylgalactosamine (GalNAc), or mannose (Man) modifies the core oligosaccharide (Moremen et al. 2012). Upon proper folding, the protein normally exits the ER and traverses the Golgi apparatus. Within the Golgi apparatus, the attached glycans are further processed by trimming and extension, creating a huge diversity of potential N-glycans (Moremen et al. 2012). While the ER is present in all compartments of the neuron, including dendrites and axons, the Golgi apparatus is usually confined to the cell body (Yadav and Linstedt 2011). This raises the question of how fast translocation of TrkB from Golgi could occur in dendrites and axonal regions that are far distant from the cell body. Golgi outputs have been identified in dendrites of rat hippocampal (Hanus and Ehlers 2008; Horton and Ehlers 2003; Mikhaylova et al. 2016; Torre and Steward 1996) and spinal motoneurons (Gardiol et al. 1999), and these sites seem to be in close association with ER structures for local synthesis of transmembrane proteins, at least in dendritic microdomains. Such regions could also contain TrkB, based on its association with the ER-Golgi intermediate compartment (ERGIC) and the cis-Golgi (Schecterson et al. 2010). Nevertheless, translocation of TrkB via ERGIC and Golgi seems unlikely to be a fast process that occurs within seconds. This raises questions on the mechanisms of how TrkB could be recruited within seconds from intracellular stores to specific domains on the cell surface.

An unconventional secretory pathway from the ER to the cell surface that is independent of the Golgi apparatus has recently been described (Hanus et al. 2016). In dendrites, hundreds of “immature” core-glycosylated proteins reach the cell surface without being further modified in the Golgi. These proteins mainly function in the context of neuronal development and synaptic plasticity (Hanus et al. 2016). This suggests that dendritic and probably also axonal proteins can be directly translocated from the ER to the cell surface. Notably, TrkB has also been identified among these “immature” core-glycosylated proteins (Hanus et al. 2016). The Trk receptor family contains nine sites for potential N-glycosylation of which four are highly conserved (Watson et al. 1999). This is in line with findings that the N-glycosylation of Trk receptors contributes to cell surface translocation in early developing cortical neurons (Puehringer et al. 2013). In these cells, TrkB is densely expressed but mostly localized in the ER so that these early cortical neurons lack sufficient TrkB cell surface expression to respond to BDNF stimulation. The cell surface translocation is initiated by Src and Fyn-induced phosphorylation of C-terminal Shc and PLCγ sites of TrkB. Thus, transactivation via Src kinases appears as an important event during early postnatal development. A similar observation was made in hippocampal neurons in which TrkB phosphorylation of Ser478 in the juxtamembrane cytoplasmatic region by cLTP-induced cyclin-dependent kinase 5 (Cdk5) activity leads to rapid TrkB recruitment to the cell surface (Zhao et al. 2009). The intracellular retention of TrkB within the ER seems to be regulated by N-glycosylation (Puehringer et al. 2013). Treatment with tunicamycin that inhibits the first step of N-glycosylation causes enhanced cell surface translocation of TrkB (Puehringer et al. 2013). In contrast to acting as a retention signal for TrkB and TrkC in early cortical precursors, N-glycosylation of TrkA on at least 9 glycosylation sites has been reported as a requirement for reaching the cell surface in PC-12 cells to respond to its ligand NGF (Schecterson et al. 2010; Watson et al. 1999). Inhibition of TrkA N-glycosylation by tunicamycin in PC12 cells led to an unglycosylated TrkA core protein that remained intracellular and showed ligand-independent autophosphorylation of the Shc binding site, without being able to induce Erk1/2 downstream signaling (Watson et al. 1999). These data indicate that TrkA requires N-glycosylation for cell surface translocation (Watson et al. 1999), while TrkB does not (Puehringer et al. 2013). Similar effects of N-glycosylation were also observed for insulin receptors that are retained intracellularly within the ER upon glucose deprivation or tunicamycin treatment, both of which result in a deglycosylated receptor of reduced size (Hwang and Frost 1999; Ronnett et al. 1984). Also, the translocation of other transmembrane proteins from the ER to the cell surface, such as the ß1 subunit of the Na-K-ATPase and occludin, are regulated by mechanisms involving N-glycosylation (Vagin et al. 2009).

Taken together, N-glycosylation appears as a modulatory mechanism for retention or export of Trk receptors from the ER. While TrkA and insulin receptors require N-glycosylation for surface export, the same condition retains TrkB in the ER, leading to the conclusion that N-glycosylation has individual effects on distinct Trk receptors. Notably, TrkB export from the ER after EGF-mediated transactivation is a very fast process and occurs within seconds. For this reason, it appears unlikely that this mechanism involves processing in the ERGIC and Golgi. This observation rather favors the idea of a direct ER export to the cell surface (Puehringer et al. 2013). However, transactivation of TrkB by GPCRs appears to follow a slower time course and seems to involve ERGIC and Golgi (Iwakura et al. 2008; Schecterson et al. 2010). More work needs to be conducted in order to understand the individual mechanisms which control Trk receptor N-glycosylation and its role for surface expression in different populations of neurons.

## Conclusion

The development and function of neuronal networks, especially correct communication and adequate responses to incoming synaptic signals, crucially depend on neurotrophin signaling. TrkB and TrkA differ in the way how they induce cell death in the absence of corresponding ligands (Nikoletopoulou et al. 2010), and TrkB has additional functions beyond promoting survival and differentiation. It modulates neuronal migration and network formation during development (Bartkowska et al. 2007; Medina et al. 2004). TrkA and TrkB also differ in the way how these receptors modulate synaptic plasticity (Bibel and Barde 2000; Poo 2001). Furthermore, the activation of TrkB and responsiveness to BDNF shifts from ligand-independent transactivation during early stages of development towards BDNF-induced receptor activation at later stages.

The regulation of TrkB cell surface expression has many advantages for the function of BDNF/TrkB signaling in the context of synaptic plasticity. Changes in subcellular cAMP could act in a highly local manner and thus fine-tune individual synapses (Maiellaro et al. 2016) in dendrites and axons, without affecting others within the same neuron. This has major consequences for shaping neuronal circuits. However, such mechanisms require fast responses, and this can only be achieved when TrkB can be recruited within a very short time from intracellular stores towards synaptic sites (Ji et al. 2005; Puehringer et al. 2013). A rapid mechanism for translocating TrkB from intracellular to defined sites at the cell surface could play a central role in such circuits, by integrating responses from firing neurons that release neurotransmitters for modulating TrkB cell surface expression via corresponding GPCRs and other neurons that release BDNF. The movement of TrkB from the ER to synaptic sites in an N-glycosylation-dependent manner, by circumventing the Golgi (Hanus et al. 2016), could provide such a rapid mechanism that alters target cell responsiveness for BDNF within seconds.

So far, it remains elusive whether the simultaneous activity of different afferents is required to induce BDNF-/TrkB-mediated LTP in such circuits. LTP then would be induced in a spatio-temporal manner only when both afferents are active. On the other side, such a scenario also would require a highly precise mechanism for removal of TrkB from the cell surface, and it is not clear whether this occurs only after ligand-induced endocytosis or also by other mechanisms. Alterations of such control mechanisms could cause aberrant synaptic plasticity and network communication and thus might be involved in numerous neuropsychiatric diseases.

## References

[CR1] Arakawa Y, Sendtner M, Thoenen H (1990). Survival effect of ciliary neurotrophic factor (CNTF) on chick embryonic motoneurons in culture: comparison with other neurotrophic factors and cytokines. J Neurosci.

[CR2] Barnes AP, Lilley BN, Pan YA, Plummer LJ, Powell AW, Raines AN, Sanes JR, Polleux F (2007). LKB1 and SAD kinases define a pathway required for the polarization of cortical neurons. Cell.

[CR3] Barres BA, Chun LL, Corey DP (1989). Calcium current in cortical astrocytes: induction by cAMP and neurotransmitters and permissive effect of serum factors. J Neurosci.

[CR4] Bartkowska K, Paquin A, Gauthier AS, Kaplan DR, Miller FD (2007). Trk signaling regulates neural precursor cell proliferation and differentiation during cortical development. Development.

[CR5] Baydyuk M, Russell T, Liao GY, Zang K, An JJ, Reichardt LF, Xu B (2011). TrkB receptor controls striatal formation by regulating the number of newborn striatal neurons. Proc Natl Acad Sci U S A.

[CR6] Becker E, Soler RM, Yuste VJ, Gine E, Sanz-Rodriguez C, Egea J, Martin-Zanca D, Comella JX (1998). Development of survival responsiveness to brain-derived neurotrophic factor, neurotrophin 3 and neurotrophin 4/5, but not to nerve growth factor, in cultured motoneurons from chick embryo spinal cord. J Neurosci.

[CR7] Berkemeier LR, Winslow JW, Kaplan DR, Nikolics K, Goeddel DV, Rosenthal A (1991). Neurotrophin-5: a novel neurotrophic factor that activates trk and trkB. Neuron.

[CR8] Bibel M, Barde YA (2000). Neurotrophins: key regulators of cell fate and cell shape in the vertebrate nervous system. Genes Dev.

[CR9] Birren SJ, Verdi JM, Anderson DJ (1992). Membrane depolarization induces p140trk and NGF responsiveness, but not p75LNGFR, in MAH cells. Science.

[CR10] Boulanger L, Poo MM (1999). Gating of BDNF-induced synaptic potentiation by cAMP. Science.

[CR11] Cabelli RJ, Hohn A, Shatz CJ (1995). Inhibition of ocular dominance column formation by infusion of NT-4/5 or BDNF. Science.

[CR12] Castellino AM, Chao MV (1996). Trans-signaling by cytokine and growth factor receptors. Cytokine Growth Factor Rev.

[CR13] Chao MV (2003). Neurotrophins and their receptors: a convergence point for many signalling pathways. Nat Rev Neurosci.

[CR14] Cheng PL, Song AH, Wong YH, Wang S, Zhang X, Poo MM (2011). Self-amplifying autocrine actions of BDNF in axon development. Proc Natl Acad Sci U S A.

[CR15] Clapham DE, Neer EJ (1993). New roles for G-protein beta gamma-dimers in transmembrane signalling. Nature.

[CR16] Crowley C, Spencer SD, Nishimura MC, Chen KS, Pitts-Meek S, Armanini MP, Ling LH, McMahon SB, Shelton DL, Levinson AD (1994). Mice lacking nerve growth factor display perinatal loss of sensory and sympathetic neurons yet develop basal forebrain cholinergic neurons. Cell.

[CR17] Davies AM, Lindsay RM (1985). The cranial sensory ganglia in culture - differences in the response of placode-derived and neural crest-derived neurons to nerve growth-factor. Dev Biol.

[CR18] Day M, Wang Z, Ding J, An X, Ingham CA, Shering AF, Wokosin D, Ilijic E, Sun Z, Sampson AR, Mugnaini E, Deutch AY, Sesack SR, Arbuthnott GW, Surmeier DJ (2006). Selective elimination of glutamatergic synapses on striatopallidal neurons in Parkinson disease models. Nat Neurosci.

[CR19] Deutch AY (2006) Striatal plasticity in parkinsonism: dystrophic changes in medium spiny neurons and progression in Parkinson’s disease. J Neural Transm Suppl:67–70 10.1007/978-3-211-45295-0_12 17017511

[CR20] Deutch AY, Colbran RJ, Winder DJ (2007). Striatal plasticity and medium spiny neuron dendritic remodeling in parkinsonism. Parkinsonism Relat Disord.

[CR21] Du X, Stull ND, Iacovitti L (1995). Brain-derived neurotrophic factor works coordinately with partner molecules to initiate tyrosine hydroxylase expression in striatal neurons. Brain Res.

[CR22] Du J, Feng L, Yang F, Lu B (2000). Activity- and Ca(2+)-dependent modulation of surface expression of brain-derived neurotrophic factor receptors in hippocampal neurons. J Cell Biol.

[CR23] Edgar D, Thoenen H (1982). Modulation of NGF-induced survival of chick sympathetic neurons by contact with a conditioned medium factor bound to the culture substrate. Brain Res.

[CR24] Gainetdinov RR, Premont RT, Bohn LM, Lefkowitz RJ, Caron MG (2004). Desensitization of G protein-coupled receptors and neuronal functions. Annu Rev Neurosci.

[CR25] Gardiol A, Racca C, Triller A (1999). Dendritic and postsynaptic protein synthetic machinery. J Neurosci.

[CR26] Gerfen CR (2006). Indirect-pathway neurons lose their spines in Parkinson disease. Nat Neurosci.

[CR27] Hamburger V (1934). The effects of wing bud extirpation on the development of the central nervous system in chick embryos. J Exp Zool.

[CR28] Hanson MG, Shen S, Wiemelt AP, McMorris FA, Barres BA (1998). Cyclic AMP elevation is sufficient to promote the survival of spinal motor neurons in vitro. J Neurosci.

[CR29] Hanus C, Ehlers MD (2008). Secretory outposts for the local processing of membrane cargo in neuronal dendrites. Traffic.

[CR30] Hanus C, Geptin H, Tushev G, Garg S, Alvarez-Castelao B, Sambandan S, Kochen L, Hafner AS, Langer JD, Schuman EM (2016) Unconventional secretory processing diversifies neuronal ion channel properties. Elife 5 10.7554/eLife.20609 PMC5077297 27677849

[CR31] Hisanaga E, Nagasawa M, Ueki K, Kulkarni RN, Mori M, Kojima I (2009). Regulation of calcium-permeable TRPV2 channel by insulin in pancreatic beta-cells. Diabetes.

[CR32] Horton AC, Ehlers MD (2003). Dual modes of endoplasmic reticulum-to-Golgi transport in dendrites revealed by live-cell imaging. J Neurosci.

[CR33] Huang EJ, Reichardt LF (2003). Trk receptors: roles in neuronal signal transduction. Annu Rev Biochem.

[CR34] Huang Y, Thathiah A (2015). Regulation of neuronal communication by G protein-coupled receptors. FEBS Lett.

[CR35] Hughes RA, Sendtner M, Thoenen H (1993). Members of several gene families influence survival of rat motoneurons in vitro and in vivo. J Neurosci Res.

[CR36] Hwang JB, Frost SC (1999). Effect of alternative glycosylation on insulin receptor processing. J Biol Chem.

[CR37] Ingham CA, Hood SH, Arbuthnott GW (1989). Spine density on neostriatal neurones changes with 6-hydroxydopamine lesions and with age. Brain Res.

[CR38] Ingham CA, Hood SH, Taggart P, Arbuthnott GW (1998). Plasticity of synapses in the rat neostriatum after unilateral lesion of the nigrostriatal dopaminergic pathway. J Neurosci.

[CR39] Iwakura Y, Nawa H, Sora I, Chao MV (2008). Dopamine D1 receptor-induced signaling through TrkB receptors in striatal neurons. J Biol Chem.

[CR40] Ji Y, Pang PT, Feng L, Lu B (2005). Cyclic AMP controls BDNF-induced TrkB phosphorylation and dendritic spine formation in mature hippocampal neurons. Nat Neurosci.

[CR41] Jia Y, Gall CM, Lynch G (2010). Presynaptic BDNF promotes postsynaptic long-term potentiation in the dorsal striatum. J Neurosci.

[CR42] Kaplan DR, Miller FD (2000). Neurotrophin signal transduction in the nervous system. Curr Opin Neurobiol.

[CR43] Kishi M, Pan YA, Crump JG, Sanes JR (2005). Mammalian SAD kinases are required for neuronal polarization. Science.

[CR44] Kreitzer AC, Malenka RC (2005). Dopamine modulation of state-dependent endocannabinoid release and long-term depression in the striatum. J Neurosci.

[CR45] Lee FS, Chao MV (2001). Activation of Trk neurotrophin receptors in the absence of neurotrophins. Proc Natl Acad Sci U S A.

[CR46] Lewin GR, Barde YA (1996). Physiology of the neurotrophins. Annu Rev Neurosci.

[CR47] Lewis SA, de Moura JL (1984). Apical membrane area of rabbit urinary bladder increases by fusion of intracellular vesicles: an electrophysiological study. J Membr Biol.

[CR48] Li JH, Palmer LG, Edelman IS, Lindemann B (1982). The role of sodium-channel density in the natriferic response of the toad urinary bladder to an antidiuretic hormone. J Membr Biol.

[CR49] Li Y, Yui D, Luikart BW, McKay RM, Li Y, Rubenstein JL, Parada LF (2012). Conditional ablation of brain-derived neurotrophic factor-TrkB signaling impairs striatal neuron development. Proc Natl Acad Sci U S A.

[CR50] Liu X, Ernfors P, Wu H, Jaenisch R (1995). Sensory but not motor neuron deficits in mice lacking NT4 and BDNF. Nature.

[CR51] Lohof AM, Ip NY, Poo MM (1993). Potentiation of developing neuromuscular synapses by the neurotrophins NT-3 and BDNF. Nature.

[CR52] Lu B (2004). Acute and long-term synaptic modulation by neurotrophins. Prog Brain Res.

[CR53] Luttrell LM (2008). Reviews in molecular biology and biotechnology: transmembrane signaling by G protein-coupled receptors. Mol Biotechnol.

[CR54] Maiellaro I, Lohse MJ, Kittel RJ, Calebiro D (2016). cAMP signals in drosophila motor neurons are confined to single synaptic boutons. Cell Rep.

[CR55] Maisonpierre PC, Belluscio L, Friedman B, Alderson RF, Wiegand SJ, Furth ME, Lindsay RM, Yancopoulos GD (1990). NT-3, BDNF, and NGF in the developing rat nervous system: parallel as well as reciprocal patterns of expression. Neuron.

[CR56] McAllister AK, Katz LC, Lo DC (1996). Neurotrophin regulation of cortical dendritic growth requires activity. Neuron.

[CR57] McAllister AK, Katz LC, Lo DC (1999). Neurotrophins and synaptic plasticity. Annu Rev Neurosci.

[CR58] McNeill TH, Brown SA, Rafols JA, Shoulson I (1988). Atrophy of medium spiny I striatal dendrites in advanced Parkinson’s disease. Brain Res.

[CR59] Medina DL, Sciarretta C, Calella AM, Von Bohlen Und Halbach O, Unsicker K, Minichiello L (2004). TrkB regulates neocortex formation through the Shc/PLCgamma-mediated control of neuronal migration. EMBO J.

[CR60] Meyer-Franke A, Kaplan MR, Pfrieger FW, Barres BA (1995). Characterization of the signaling interactions that promote the survival and growth of developing retinal ganglion cells in culture. Neuron.

[CR61] Meyer-Franke A, Wilkinson GA, Kruttgen A, Hu M, Munro E, Hanson MG, Reichardt LF, Barres BA (1998). Depolarization and cAMP elevation rapidly recruit TrkB to the plasma membrane of CNS neurons. Neuron.

[CR62] Mikhaylova M, Bera S, Kobler O, Frischknecht R, Kreutz MR (2016). A dendritic Golgi satellite between ERGIC and Retromer. Cell Rep.

[CR63] Moremen KW, Tiemeyer M, Nairn AV (2012). Vertebrate protein glycosylation: diversity, synthesis and function. Nat Rev Mol Cell Biol.

[CR64] Morris AJ, Scarlata S (1997). Regulation of effectors by G-protein alpha- and beta gamma-subunits. Recent insights from studies of the phospholipase c-beta isoenzymes. Biochem Pharmacol.

[CR65] Murer H, Biber J (1996). Molecular mechanisms of renal apical Na/phosphate cotransport. Annu Rev Physiol.

[CR66] Nikoletopoulou V, Lickert H, Frade JM, Rencurel C, Giallonardo P, Zhang L, Bibel M, Barde YA (2010). Neurotrophin receptors TrkA and TrkC cause neuronal death whereas TrkB does not. Nature.

[CR67] Oppenheim RW, Yin QW, Prevette D, Yan Q (1992). Brain-derived neurotrophic factor rescues developing avian motoneurons from cell death. Nature.

[CR68] Plotkin JL, Day M, Peterson JD, Xie Z, Kress GJ, Rafalovich I, Kondapalli J, Gertler TS, Flajolet M, Greengard P, Stavarache M, Kaplitt MG, Rosinski J, Chan CS, Surmeier DJ (2014). Impaired TrkB receptor signaling underlies corticostriatal dysfunction in Huntington’s disease. Neuron.

[CR69] Poo MM (2001). Neurotrophins as synaptic modulators. Nat Rev Neurosci.

[CR70] Puehringer D, Orel N, Luningschror P, Subramanian N, Herrmann T, Chao MV, Sendtner M (2013). EGF transactivation of Trk receptors regulates the migration of newborn cortical neurons. Nat Neurosci.

[CR71] Purves D, Lichtman J (1985). Chapter 6: neuronal death during development. Principles of neural development.

[CR72] Purves D, Lichtman J (1985). Chapter 7: trophic effects of targets on neurons. Principles of neural development.

[CR73] Rauskolb S, Zagrebelsky M, Dreznjak A, Deogracias R, Matsumoto T, Wiese S, Erne B, Sendtner M, Schaeren-Wiemers N, Korte M, Barde YA (2010). Global deprivation of brain-derived neurotrophic factor in the CNS reveals an area-specific requirement for dendritic growth. J Neurosci.

[CR74] Ronnett GV, Knutson VP, Kohanski RA, Simpson TL, Lane MD (1984). Role of glycosylation in the processing of newly translated insulin proreceptor in 3T3-L1 adipocytes. J Biol Chem.

[CR75] Ruberti F, Capsoni S, Comparini A, Di Daniel E, Franzot J, Gonfloni S, Rossi G, Berardi N, Cattaneo A (2000). Phenotypic knockout of nerve growth factor in adult transgenic mice reveals severe deficits in basal forebrain cholinergic neurons, cell death in the spleen, and skeletal muscle dystrophy. J Neurosci.

[CR76] Scannevin RH, Huganir RL (2000). Postsynaptic organization and regulation of excitatory synapses. Nat Rev Neurosci.

[CR77] Schecterson LC, Hudson MP, Ko M, Philippidou P, Akmentin W, Wiley J, Rosenblum E, Chao MV, Halegoua S, Bothwell M (2010). Trk activation in the secretory pathway promotes Golgi fragmentation. Mol Cell Neurosci.

[CR78] Schwartz GJ, Al-Awqati Q (1986). Regulation of transepithelial H+ transport by exocytosis and endocytosis. Annu Rev Physiol.

[CR79] Sendtner M, Holtmann B, Kolbeck R, Thoenen H, Barde YA (1992). Brain-derived neurotrophic factor prevents the death of motoneurons in newborn rats after nerve section. Nature.

[CR80] Shelly M, Cancedda L, Heilshorn S, Sumbre G, Poo MM (2007). LKB1/STRAD promotes axon initiation during neuronal polarization. Cell.

[CR81] Shelly M, Cancedda L, Lim BK, Popescu AT, Cheng PL, Gao H, Poo MM (2011). Semaphorin3A regulates neuronal polarization by suppressing axon formation and promoting dendrite growth. Neuron.

[CR82] Shen W, Flajolet M, Greengard P, Surmeier DJ (2008). Dichotomous dopaminergic control of striatal synaptic plasticity. Science.

[CR83] Shen W, Plotkin JL, Francardo V, Ko WK, Xie Z, Li Q, Fieblinger T, Wess J, Neubig RR, Lindsley CW, Conn PJ, Greengard P, Bezard E, Cenci MA, Surmeier DJ (2016). M4 muscarinic receptor signaling ameliorates striatal plasticity deficits in models of L-DOPA-induced dyskinesia. Neuron.

[CR84] Sheng M (2001). Molecular organization of the postsynaptic specialization. Proc Natl Acad Sci U S A.

[CR85] Shimada A, Mason CA, Morrison ME (1998). TrkB signaling modulates spine density and morphology independent of dendrite structure in cultured neonatal Purkinje cells. J Neurosci.

[CR86] Sui WH, Huang SH, Wang J, Chen Q, Liu T, Chen ZY (2015). Myosin Va mediates BDNF-induced postendocytic recycling of full-length TrkB and its translocation into dendritic spines. J Cell Sci.

[CR87] Tartaglia N, Du J, Tyler WJ, Neale E, Pozzo-Miller L, Lu B (2001). Protein synthesis-dependent and -independent regulation of hippocampal synapses by brain-derived neurotrophic factor. J Biol Chem.

[CR88] Thoenen H, Barde YA, Edgar D (1981). The role of nerve growth factor (NGF) and related factors for the survival of peripheral neurons. Adv Biochem Psychopharmacol.

[CR89] Torre ER, Steward O (1996). Protein synthesis within dendrites: glycosylation of newly synthesized proteins in dendrites of hippocampal neurons in culture. J Neurosci.

[CR90] Tyler WJ, Pozzo-Miller LD (2001). BDNF enhances quantal neurotransmitter release and increases the number of docked vesicles at the active zones of hippocampal excitatory synapses. J Neurosci.

[CR91] Vagin O, Kraut JA, Sachs G (2009). Role of N-glycosylation in trafficking of apical membrane proteins in epithelia. Am J Physiol Renal Physiol.

[CR92] Villalba RM, Smith Y (2010). Striatal spine plasticity in Parkinson’s disease. Front Neuroanat.

[CR93] Villalba RM, Lee H, Smith Y (2009). Dopaminergic denervation and spine loss in the striatum of MPTP-treated monkeys. Exp Neurol.

[CR94] Wade JB (1986). Role of membrane fusion in hormonal regulation of epithelial transport. Annu Rev Physiol.

[CR95] Wang T, Xie K, Lu B (1995). Neurotrophins promote maturation of developing neuromuscular synapses. J Neurosci.

[CR96] Watson FL, Porcionatto MA, Bhattacharyya A, Stiles CD, Segal RA (1999). TrkA glycosylation regulates receptor localization and activity. J Neurobiol.

[CR97] Wiese S, Jablonka S, Holtmann B, Orel N, Rajagopal R, Chao MV, Sendtner M (2007). Adenosine receptor A2A-R contributes to motoneuron survival by transactivating the tyrosine kinase receptor TrkB. Proc Natl Acad Sci U S A.

[CR98] Xia ZG, Refsdal CD, Merchant KM, Dorsa DM, Storm DR (1991). Distribution of mRNA for the calmodulin-sensitive adenylate cyclase in rat brain: expression in areas associated with learning and memory. Neuron.

[CR99] Yadav S, Linstedt AD (2011) Golgi positioning. Cold Spring Harb Perspect Biol 3 10.1101/cshperspect.a005322 PMC3101843 21504874

[CR100] Yan Q, Elliott J, Snider WD (1992). Brain-derived neurotrophic factor rescues spinal motor neurons from axotomy-induced cell death. Nature.

[CR101] Yan Z, Feng J, Fienberg AA, Greengard P (1999). D(2) dopamine receptors induce mitogen-activated protein kinase and cAMP response element-binding protein phosphorylation in neurons. Proc Natl Acad Sci U S A.

[CR102] Yao X, Karam SM, Ramilo M, Rong Q, Thibodeau A, Forte JG (1996). Stimulation of gastric acid secretion by cAMP in a novel alpha-toxin-permeabilized gland model. Am J Phys.

[CR103] Yudowski GA, Puthenveedu MA, Leonoudakis D, Panicker S, Thorn KS, Beattie EC, von Zastrow M (2007). Real-time imaging of discrete exocytic events mediating surface delivery of AMPA receptors. J Neurosci.

[CR104] Zaja-Milatovic S, Milatovic D, Schantz AM, Zhang J, Montine KS, Samii A, Deutch AY, Montine TJ (2005). Dendritic degeneration in neostriatal medium spiny neurons in Parkinson disease. Neurology.

[CR105] Zhai S, Tanimura A, Graves SM, Shen W, Surmeier DJ (2018). Striatal synapses, circuits, and Parkinson’s disease. Curr Opin Neurobiol.

[CR106] Zhai S, Shen W, Graves SM, Surmeier DJ (2019). Dopaminergic modulation of striatal function and Parkinson’s disease. J Neural Transm (Vienna).

[CR107] Zhang Y, Moheban DB, Conway BR, Bhattacharyya A, Segal RA (2000). Cell surface Trk receptors mediate NGF-induced survival while internalized receptors regulate NGF-induced differentiation. J Neurosci.

[CR108] Zhao L, Sheng AL, Huang SH, Yin YX, Chen B, Li XZ, Zhang Y, Chen ZY (2009). Mechanism underlying activity-dependent insertion of TrkB into the neuronal surface. J Cell Sci.

